# Harnessing novel genetic markers for scald resistance from gene bank spring barley genotypes

**DOI:** 10.1186/s12870-025-06813-2

**Published:** 2025-06-11

**Authors:** Su Myat Noe, Johanna Åstrand, Mustafa Zakieh, Pawan Kumar Singh, Eva Johansson, Aakash Chawade

**Affiliations:** 1 Department of Plant Breeding, Swedish University of Agricultural Sciences, Alnarp, Sweden; 2 Lantmännen Lantbruk, Svalöv, Sweden; 3 International Maize and Wheat Improvement Center (CIMMYT), Veracruz 56237, El Batan, Texcoco, 56237 Mexico

**Keywords:** GWAS, Barley, Scald, AUDPC, Biotic stress

## Abstract

**Background:**

Scald caused by *Rhynchosporium graminicola* is a common foliar disease affecting barley production worldwide. Identifying and utilizing scald resistance genes and quantitative trait loci (QTL) to develop barley cultivars with durable and effective resistance to scald is crucial.

**Results:**

In the present study, we evaluated 275 spring barley genotypes together with 4 commercial check cultivars under controlled conditions and examined the underlying genetics of scald resistance in these genotypes. A significant genetic variation (*P* value < 0.0001) for scald resistance was observed among the tested barley germplasms. A genome-wide association study (GWAS) identified eight markers‒trait associations (MTAs) forming seven QTL located on chromosomes 3H, 6H, and 7H, of which three are novel. The allelic effects of these MTAs were further examined, and favorable alleles associated with scald resistance were identified.

**Conclusions:**

The identification of QTL for scald resistance, along with favorable allele identification, will be crucial for marker-assisted breeding programs. These findings will facilitate the development of new scald-resistant cultivars and contribute to the sustainability of barley production. Further studies, such as fine-mapping of candidate genes within these identified QTL regions, will help to narrow down the potential causative genetic variants and understand their functional effects on scald resistance.

**Supplementary Information:**

The online version contains supplementary material available at 10.1186/s12870-025-06813-2.

## Background

Barley (*Hordeum vulgare* L.) is an annual grass in the Poaceae family. Despite its adaptability to various environments, barley is cultivated primarily in temperate regions [1]. Globally, barley is the fifth most produced cereal crop in terms of production acreage [2]. In 2020/2021, 160 million metric tons of barley were produced, with the European Union being the most productive region with 53 million metric tons [3].

Improving crop yield is a primary objective of plant breeding programs; however, reaching the full yield potential is severely prohibited by abiotic (such as drought and temperature) and biotic (pests and diseases) constraints [4–6]. Plant diseases such as net blotch, scald, leaf blotch, brown rust, and powdery mildew significantly affect grain yield, quality and the biomass harvested for feed [7]. Among these diseases, scald can cause 30% yield loss and reduce grain quality [8]. The causal pathogen of scald disease is *Rhynchosporium graminicola* (formerly known as *R. commune*), a hemibiotroph fungus. It overwinters on plant residues, seeds, and soil, where the first two serve as the primary sources of inoculum and sporulation [9].

Eleven major resistance genes and several quantitative trait loci (QTL) associated with scald resistance have been identified and mapped as summarized in Supplementary Table S1. Among these, *Rrs1* was the first scald resistance gene discovered in barley and *Rrs18* is the most recent scald resistance gene, the latter has been mapped on Chr 6H. Additional examples are *Rrs4* mapped on Chr 3H in cultivated barley and *Rrs13* which was reported in an interspecific cross between *H. vulgare* and *H. spontaneum* [10–21]. Although multiple resistance genes for scald disease have been discovered and subsequently used in breeding programs, the pathogen evolve and develop virulence to overcome resistance genes. The defense system in plants are overcome due to several biotic and abiotic selection pressures, such as mutations (gene-for-gene interactions between pathogens and host plants), climate change, and fungicide application [22]. One way to delay the breakdown of disease resistance is by broadening the genetic basis of host resistance through the pyramiding of resistance genes together with QTL with minor- to moderate effect, which may also result in more durable and effective resistance to scald disease [23]. Therefore, the discovery of qualitative and quantitative resistance genes for scald disease resistance from various genetic resources, such as diverse germplasms from gene bank, is crucial.

As of 2019, 1,750 gene banks worldwide have collected 7.5 million germplasms for several plant species, including landraces, wild relatives, mutants, and genetic resources, which are conserved in different forms, such as seeds and other plant parts, including shoots and pollens [24]. Landraces and wild relatives likely exhibit tolerance and resistance to biotic stresses, providing potential benefits for modern cultivars. Recent advances in genotyping technologies and reduced costs associated with sequencing allow researchers to effectively explore diversity in gene bank accessions. Genome-wide association studies (GWAS) have emerged as a powerful tool in identifying the genetic basis of complex traits, including disease resistance in barley. For example, a GWAS conducted using a worldwide barley collection, including 277 landraces, identified 15 QTL for net form net blotch (NFNB) (*Pyrenophora teres* f. *teres*) resistance, four of which were newly reported QTL [25]. Another association mapping study used wild and landrace barley populations collected across Türkiye and detected four and ten QTL associated with SFNB spot form net blotch (SFNB) and NFNB, respectively [26]. A GWAS study identified two new major QTL for powdery mildew resistance from a mixture of 696 barley accessions where more than half of the mixture consisted of wild type and landraces [27]. In addition, an association study using Ethiopian landraces identified 17 marker-trait associations (MTAs) across seven chromosomes associated with barley scald disease [28]. Moreover, a GWAS analysis was conducted with 131 Scottish Bere barley landraces resulting in the detection of a number of genomic regions associated with scald resistance and among them, four QTL were novel of which candidate gene were identified [29]. A recent genetic association study identified 22 QTL, some of which some were novel and associated with scald resistance. The synchronized use of advanced technologies like GWAS and the rich genetic diversity enables precise identification of resistance genes and QTL, enabling targeted breeding strategies to enhance crop resilience against biotic stresses [9].

The current study utilized a diverse panel of 275 barley genotypes from the NordGen gene bank to evaluate scald resistance by artificial inoculation under greenhouse conditions. The objectives of the study were to i) evaluate the scald resistance of the tested gene bank accessions, ii) examine the genetic makeup and patterns of genetic association within the gene bank accessions, iii) identify QTL associated with scald resistance from the current barley germplasm, and iv) compare these with previous studies to identify novel findings and resources for breeding programs.

## Results

### Phenotypic variation among the tested barley accessions

Pearson correlation coefficients were computed to assess the consistency between the two experiments. A moderate correlation (*r* = 0.61) was observed between the two experiments. Meanwhile, the correlations between the BLUE values (estimated from AUDPC data across both experiments) and individual AUDPC observations were strong, with *r* = 0.87 for BLUE vs. AUDPC in experiment 1 and *r* = 0.90 for BLUE vs. AUDPC in experiment 2. These strong correlations confirmed that the BLUE values reliably capture genotype performance based on AUDPC.

**Fig. 1:**
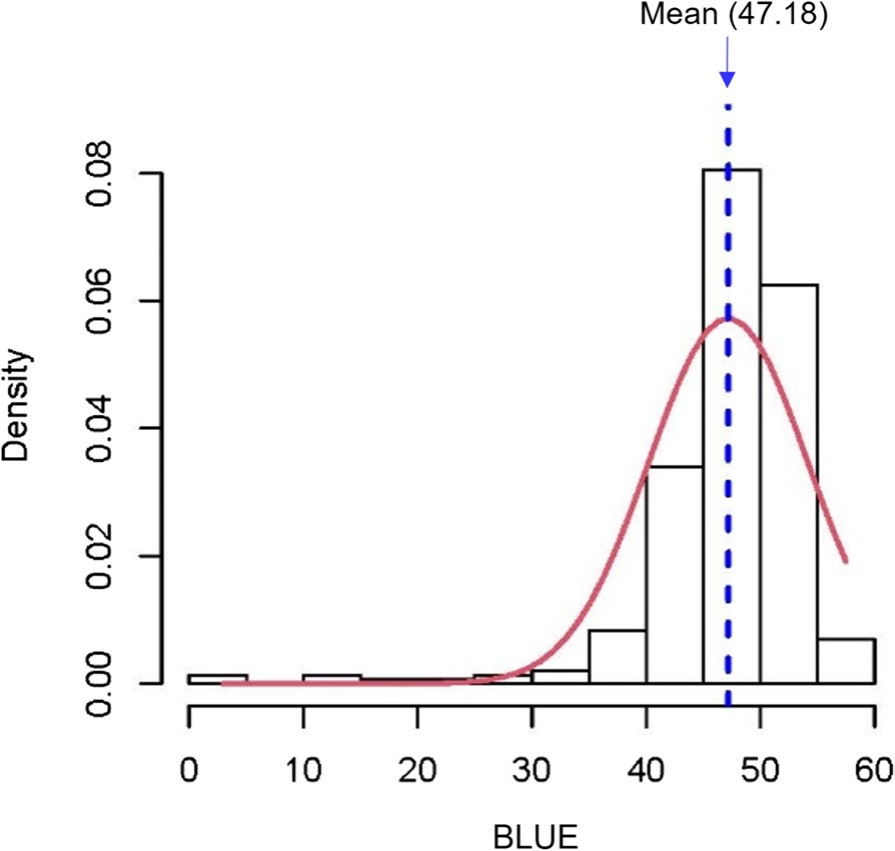
Distribution of best linear unbiased estimation (BLUE) values for scald disease in the spring barley population. The mean BLUE of the total population was 47.18. The population was skewed towards higher infection scores indicating high susceptibility in the germplasm

**Fig. 2:**
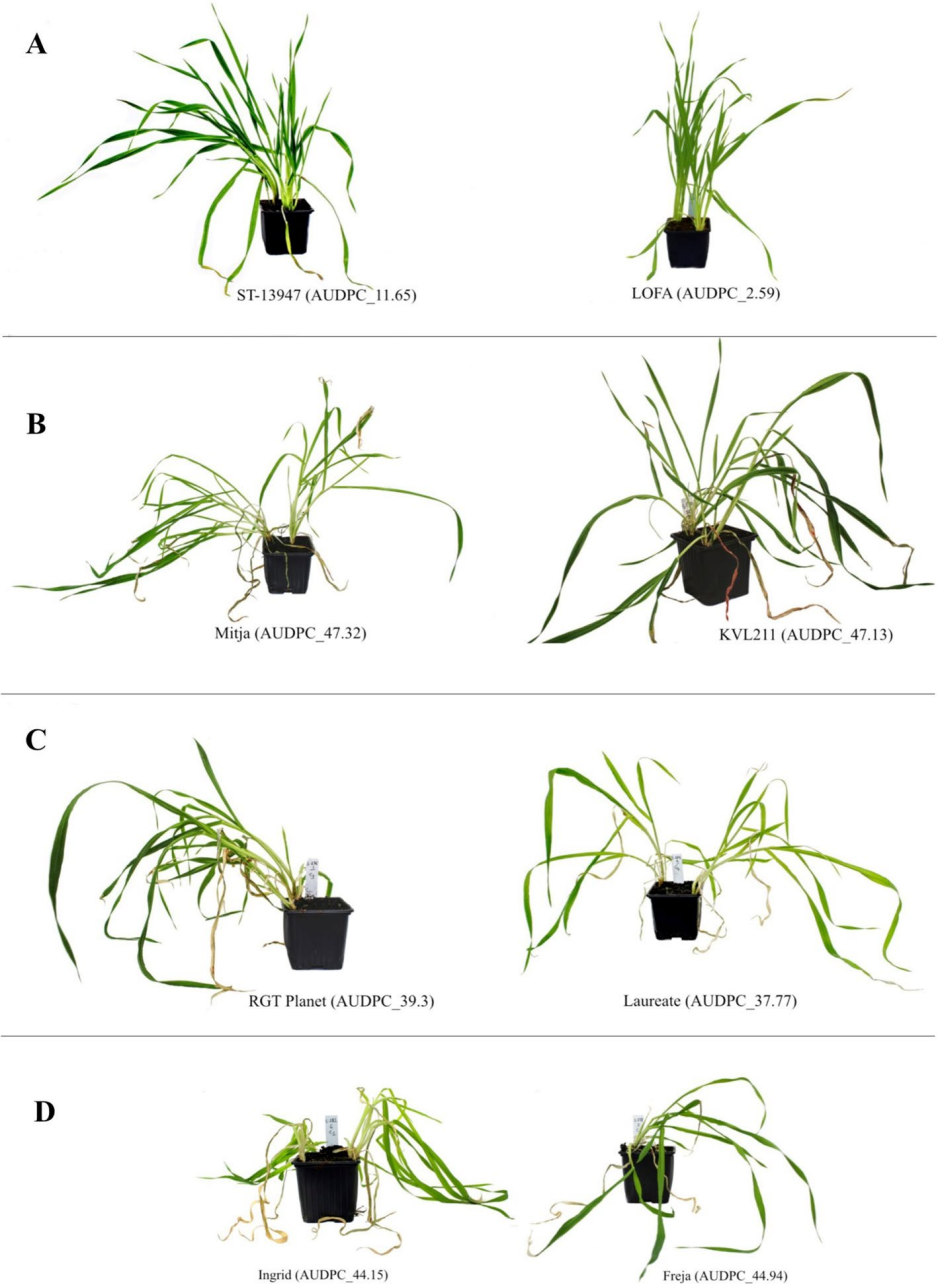
Barley genotypes with low or high AUDPC associated with resistance and susceptibility against scald disease and the check cultivars during third scoring (17 DAI). Cultivars having low BLUE values; ST-13947 (left) and Lofa (right) with BLUE of 11.65 and 2.59 respectively; **B** Genotypes having high BLUE values; Mitja (left) and KVL211 (right) with BLUE of 47.32 and 47.13 respectively; **C** Moderately resistant check cultivars; RGT Planet (left) and Laureate (right) with BLUE of 39.3 and 37.77, respectively; **D** Susceptible check cultivars; Ingrid (left) and Freja (right) with BLUE of 44.15 and 44.94 respectively

The values for BLUE ranged from 2.59 to 51.96, indicating a left-skewed frequency distribution where lower values suggest greater genotype resistance. One hundred and sixty-five genotypes had a BLUE greater than the BLUE mean (42.6), while the remaining 113 genotypes had a lower BLUE than the mean (Fig. 1). The susceptible check cultivars Freja and Ingrid attained BLUE values of 44.94 and 44.15 (above the mean), respectively. In contrast, the moderately resistant check cultivars Laureate and RGT Planet achieved BLUE values of 37.77 and 39.3 (below the mean), respectively. Analysis of variance revealed a high significant phenotypic variation among the studied genotypes (*P* value < 0.0001) and a broad-sense heritability of 0.73 was observed. The calculated BLUE values are shown in Supplementary Table S2. Examples of the phenotypic differences among the tested barley genotypes, along with the resistant and susceptible check cultivars, are shown in Fig. 2A-D. Figure 2A illustrates two genotypes with low BLUE, LOFA and ST-13947, while Fig. 2B shows two genotypes with high BLUE, Mitja and KVL211. Figure 2C illustrates the moderately resistant checks—Laureate and RGT planet—and Fig. 2D demonstrates the susceptible checks Ingrid and Freja.

### Linkage disequilibrium

**Fig. 3:**
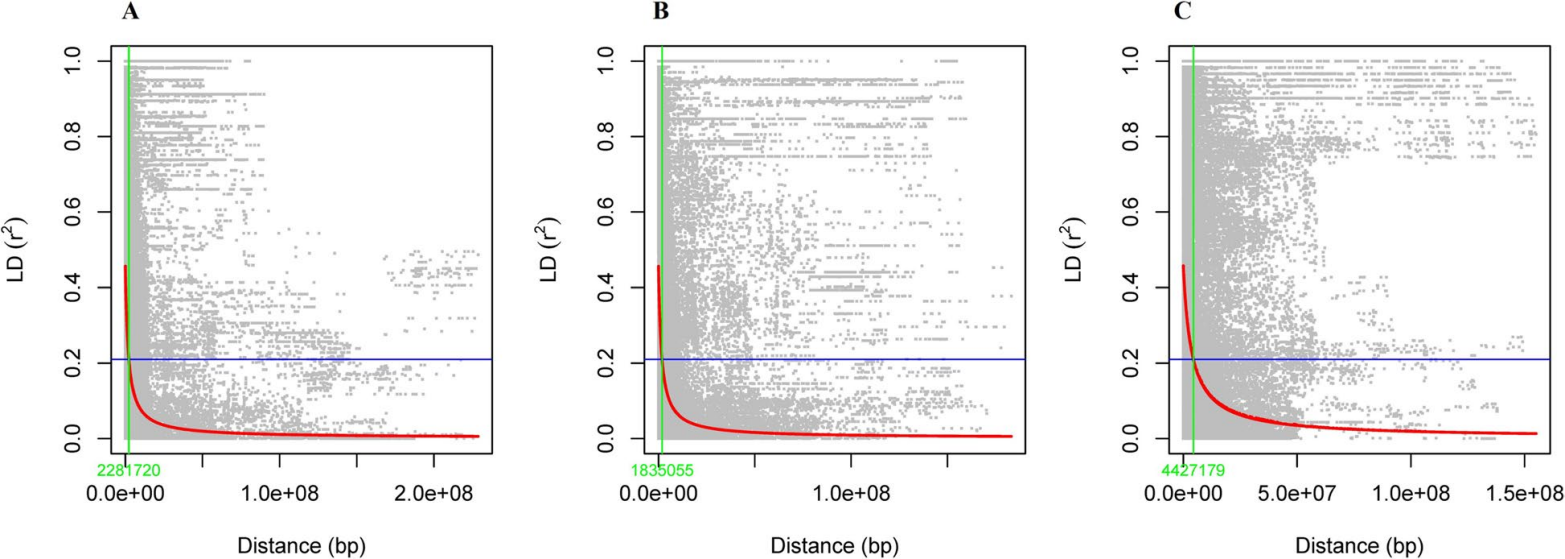
Scatter plots (**A**, **B** and **C**) illustrating the chromosome-wide linkage disequilibrium (LD) decay for Chr 3H, 6H, and 7H, where peak markers were identified respectively. The plot displays r^2^ values against physical distance (in base pairs). The red curve represents the smoothing spline regression model fitted to the LD decay, with a maximum LD decay of 0.46 observed for all chromosomes. A horizontal blue line marks the half-decay r^2^ value (0.23 for all chromosomes), and a green vertical line indicates the distance between marker pairs (1,181,720 bp, 4,427,179 bp, and 1,835,055 bp for chromosomes 3H, 6H, and 7H, respectively). The intersection between the green line and the blue half-decay line corresponds to the point where LD decay reaches half its maximum value

Linkage disequilibrium (LD) decay was estimated from 10,151 SNP markers and resulted in a total number of marker pairs on each chromosome varying from 48,522 (Chr 3H) to 96,216 (Chr 5H), with a total of 496,319 marker pairs in the whole genome. The average correlation coefficient (r^2^) across the seven barley chromosomes ranged from 0.15 to 0.23, while the overall genome-wide r^2^ value was 0.17. Overall, 61% of the total marker pairs across the whole genome were in significant LD, with an average r^2^ of 0.17 at the 0.001 significance level (Table 1). The LD half decay in kilobase pairs (bp) for each chromosome varied from 1,449,455 bp (Chr 1H) to 4,427,179 bp (Chr 6H) (Fig. 3; Supplementary Fig. 2), and at the whole-genome level, the maximum LD decay was observed at a r^2^ value of 0.46 and reached its half decay (2,083,171 bp) when r^2^ reached 0.0.21 (Supplementary Fig. 2).

**Table 1:** Summary of linkage disequilibrium analysis of the 10,151 qualified SNP markers across seven chromosomes and the entire barley genome

Chromosome	Total number of marker pairs	Avg r^2^ for total marker pairs	No. of sig. marker pairs and percent of sig. Markers (*p* < = 0.001) *	Avg r^2^ for sig. pairs (*p* < = 0.001)	LD half-decay (bp) **
Chr 1H	50,879	0.15	27,721 (54)	0.15	1,449,455 (1.4)
Chr 2H	73,660	0.16	43,466 (59)	0.16	1,487,502 (1.5)
Chr 3H	79,269	0.18	48,675 (61)	0.18	2,281,720 (2.3)
Chr 4H	48,522	0.17	28,066 (58)	0.17	2,751,740 (2.8)
Chr 5H	96,216	0.18	60,977 (63)	0.18	2,009,142 (2.0)
Chr 6H	71,913	0.23	48,422 (67)	0.23	4,427,179 (4.4)
Chr 7H	75,867	0.17	47,473 (63)	0.17	1,835,055 (1.8)
Whole genome	496,319	0.21	300,613 (61)	0.21	2,083,171 (2.1)

^*^ Number in brackets represents the percentage of significant marker pairs on each chromosome as well as at the whole-genome level

^**^ Number in brackets represents the LD half-decay in mega base pairs (Mbp)

### GWAS to identify QTL associated with scald resistance

**Fig. 4:**
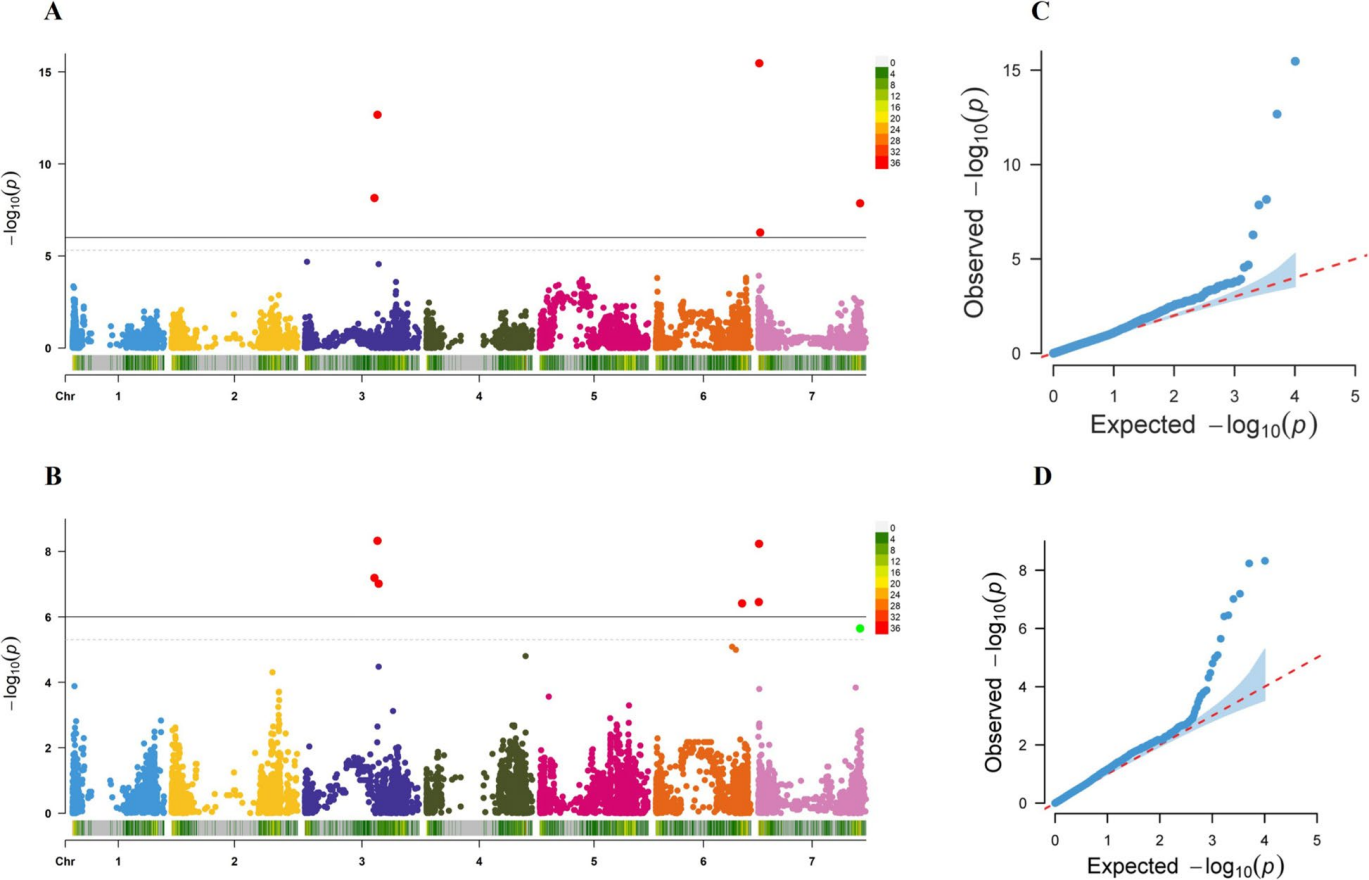
The GWAS results were based on two statistical models (BLINK and FarmCPU) using 279 spring barley accession genotyped with 10,151 SNP markers. The Manhattan plots and QQ plots show the marker trait associations from the (**A** and **C**) BLINK and the (**B** and **D**) FarmCPU model. The X-axis of Figs. 5 (**A**) and (**B**) show the chromosomes of barley altogether with the SNP density on each chromosome while the Y-axis represents -log10 (p) values. Again, on the same pictures, the dotted line and block line represented the significance threshold at 0.05 and the Bonferroni correction (5.0 × 10e^−6^), respectively. Five and six SNPs were detected above the Bonferroni threshold for BLINK and FarmCPU respectively

GWAS was conducted utilizing BLUE as phenotypic data and 10,151 SNP markers as genotypic data using two multi-locus models, BLINK and FarmCPU. These models together identified eight markers‒trait associations (MTAs) corresponding to seven QTL (Table 2; Fig. 4A-D)—three located on Chr 3H, one on Chr 6H, and three on Chr 7H. The BLINK model identified five MTAs (Fig. 4A and C), which accounted for 54.4% of the total phenotypic variance. On the other hand, the FarmCPU model identified six MTAs (Fig. 4B and D), which explained 33.9% of the phenotypic variation in the tested population (Table 2). The first QTL identified on Chr 3H was QTL_3H_1 at 424.23 Mbp, and the second QTL (QTL_3H_2) was located 17.9 Mbp away from the first QTL. The third detected QTL (QTL_3H_3) was located at 450.44 Mbp (26.2 Mbp and 8.23 Mbp away from the first and second QTL, respectively). On Chr 6H, a single QTL (QTL_6H) located at 527.21 Mbp was detected. Three QTL were detected on Chr 7H: QTL_7H_1 (5.4 Mbp), QTL_7H_2 (11.1 Mbp) and QTL_7H_3 (621.55 Mbp) (Fig. 4). Significant markers were assigned to the QTL based on their chromosomal LD half-decay estimates (1.4—4.4 Mbp across seven chromosomes) [30].

**Table 2:** List of significant SNP markers associated with scald resistance in the tested barley germplasms. Two statistical models, BLINK and FarmCPU, were used to identify eight significant SNP markers

QTL	SNP marker	Chr	Allele^1^	Pos. (Mbp)	MAF	P. value	Effect	PVE (%)	No. of MTA	Statistical Model
QTL_3H_1	BOPA1_1977-1385	3	G/**A**	424.23	0.05	7.05E-09	5.65^***^	17.9	1	BLINK
						6.42E-08	4.99^***^	18.6		FarmCPU
QTL_3H_2	JHI-Hv50k-2016–183215	3	A**/G**	442.21	0.133	4.75E-09	−2.91^***^	6.2	1	FarmCPU
						2.12E-13	−4.2^***^	9		BLINK
QTL_3H_3	BOPA1_2338-1572	3	G/**A**	450.44	0.108	9.68E-08	−1.67^ ns^	0.9	1	FarmCPU
QTL_6H	BOPA1_4146-1154	6	**A**/G	527.21	0.281	2.85E-06	1.65^ ns^	0.6	1	FarmCPU
QTL_7H_1	BOPA2_12_20201	7	G/**A**	4.58	0.081	5.81E-09	4.00^***^	4.9	1	FarmCPU
	JHI-Hv50k-2016–441289	7	C/**T**	5.4	0.059	3.37E-16	−6.2^***^	17.3	1	BLINK
QTL_7H_2	JHI-Hv50k-2016–445855	7	T/**G**	11.1	0.065	5.32E-07	−3.7^ ns^	6.9	1	BLINK
QTL_7H_3	SCRI_RS_184902	7	G/**A**	621.55	0.197	2.25E-06	−1.89^ ns^	2.7	1	FarmCPU
						1.38E-08	−2.46^ ns^	3.3		BLINK

*QTL* Quantitative Trait Loci, *Chr* Chromosome, *Pos.* Position, *MAF* Minor Allele Frequency, *PVE* Phenotype Variance Explained (%), *Effect* Allelic effect on the phenotypic variation (the allele listed first represents the major allele, while the allele written in bold signifies the favorable allele)

^1^This is an explanation for the significant difference between favorable and unfavorable alleles over the observed phenotype. ***, *P* < 0.0001; ns, not significant

**Fig. 5:**
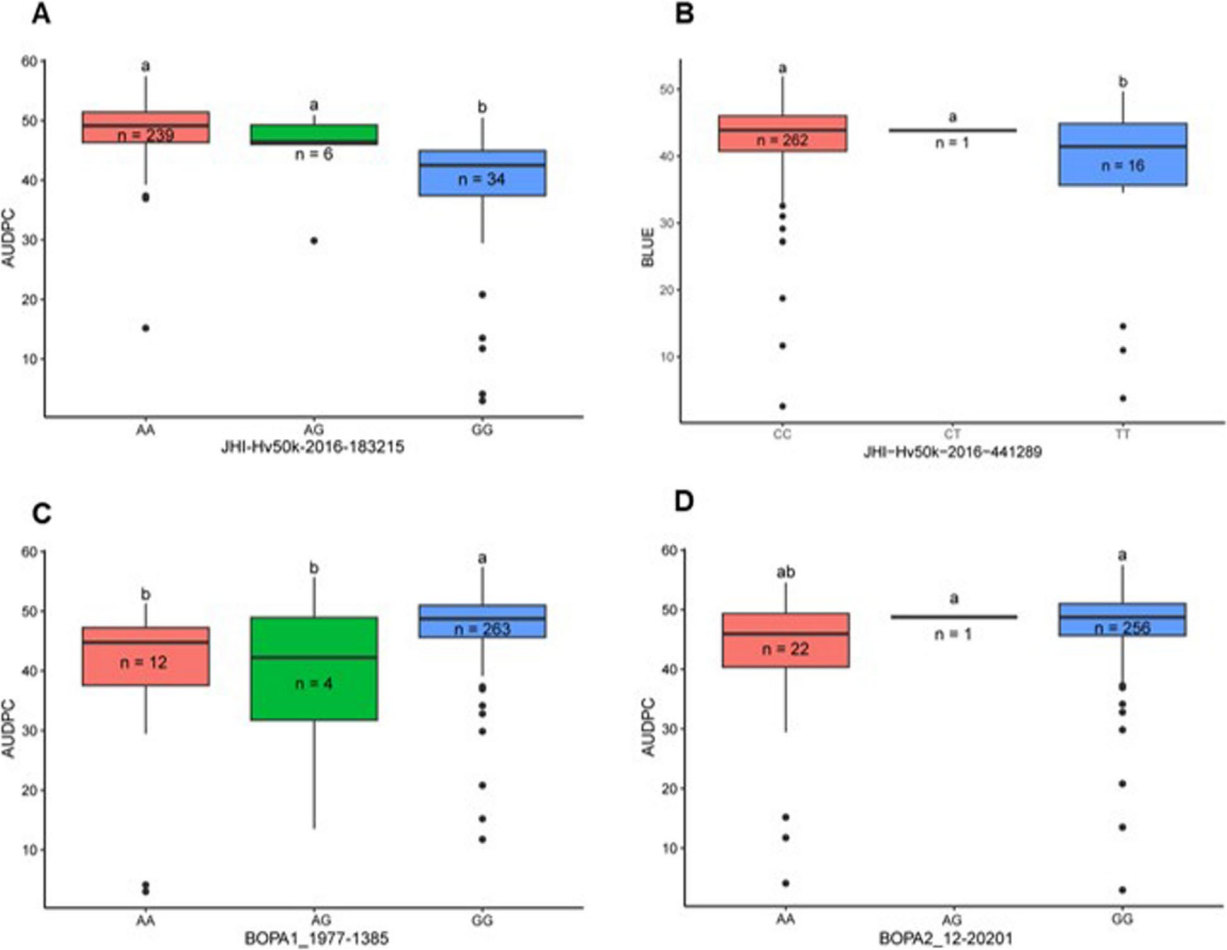
Boxplots showing the relationship between the favorable and unfavorable alleles of the significant SNPs and the phenotype of scald resistance (BLUE) in 279 barley genotypes. The impact of the beneficial and non-beneficial alleles of SNPs JHI-Hv50k-2016–183215, JHI-Hv50k-2016–441289 and BOPA1_1977-1385 and BOPA2_12_20201 are visualized in Fig. **A**-**D**. Tukey's HSD (honestly significant difference) test was performed to establish the significance level by comparing the allelic composition of the barley germplasms

After identifying QTL, we further examined the allelic effect on the observed BLUE by performing an additional post hoc test (Tukey's HSD test). The test revealed significant allelic effects for QTL_3H_1, QTL_3H_2, and QTL_7H_1 (Fig. 5A-D). No significant allelic effect was detected for the remaining QTL (Supplementary Fig. 3). QTL_3H_1 and QTL_3H_2 were detected in both the BLINK and FarmCPU models, while QTL_7H_1 was associated with two markers, each of which were detected either in the BLINK or FarmCPU models (Table 2).

Following the validation of the QTL detected in our study, we examined the tested genotypes for favorable alleles and analyzed the optimal allelic combinations of the MTAs associated with these QTL. Based on this analysis, we observed 15 allelic combinations, as shown in Supplementary Table S3. Among them, we focused on ten homozygous combinations since they provided a clearer insight into allele effects. One genotype possessed all the favorable alleles and achieved a BLUE value of 3.78. In contrast, 214 genotypes did not contain any favorable allele, and their BLUE values ranged between 32.56 and 51.96. Additionally, 32 genotypes contained a favorable allele from one of the significant MTAs, and their BLUE values ranged from 14.56 to 49.68. Twenty genotypes contained two favorable alleles from either two of the four significant MTAs, while two genotypes had three favorable alleles, resulting in BLUE values of 10.98 and 27.18, respectively.

## Methods

### Plant materials

A total of 275 spring barley accessions provided by the Nordic gene bank NordGen (https://www.nordgen.org/), along with four commercial check cultivars, were evaluated for scald resistance under greenhouse conditions. The majority of the gene bank germplasms were Swedish landraces. Among the tested cultivars, Freja and Ingrid were susceptible to scald, while Laureate and RGT Planet are known as moderately resistant cultivars. In this study, we conducted two experiments using an augmented block design. In each experiment, the gene bank materials were divided into 14 blocks, each containing a repetition of all four commercial checks and a random subset of the gene bank materials. Four to six seeds per genotype per pot (8 × 8x9 cm in size) were sown in both experiments and were used for disease evaluation.

### Inoculation

The *R. graminicola* pathogen was collected from spring barley fields in Southern Sweden near Ystad in 2022. The specific pathotype of the collected pathogen was unknown. The inoculum was multiplied under in vitro conditions by following an optimized protocol from the Department of Plant Breeding, SLU [31]. The *R. graminicola* mycelia were initially grown on water agar media (15 g of agar per liter of distilled water) for two weeks and then transferred to plates with CZV8 CYM media (Supplementary Table S4). The plates were incubated under dark conditions at 17–18 °C for two weeks until mycelial growth reached 1–3 cm in diameter. The mycelium was then transferred to wheat germ agar media (100 g of wheat germ (Risenta)and 15 g of agar per liter of distilled water). The cultured plates were incubated under dark conditions at 17–18 °C for two weeks to induce *R. graminicola* sporulation. Afterward, the conidia were collected by water flooding the plate and scrapped forcefully with a fine paintbrush. Then, the inoculum suspension was prepared at a concentration of 1.35 × 10^6^ conidia/ml using tap water and 0.02% of the surfactant Tween® 20. Inoculation was conducted at the third leaf development stage (Zadoks stage 13) by evenly spraying the plant leaves with a hand spray until the plants ran off. Immediately after inoculation, the plants were transferred to an incubation room with a relative humidity (RH) of 100% and kept under dark conditions for 72 h at 16–17 °C. Three days after inoculation, the growth conditions were adjusted to 75% RH while maintaining a 16/8 h light/dark cycle without altering the temperature.

### Disease scoring and phenotypic data analysis

Disease evaluation was carried out at 11 days post inoculation (DPI) when the initial symptoms, such as water-soaked lesions, developed on the leaf surface of the inoculated second and third leaves. Subsequent scoring was conducted at 14 and 17 DPI.

**Fig. 6:**
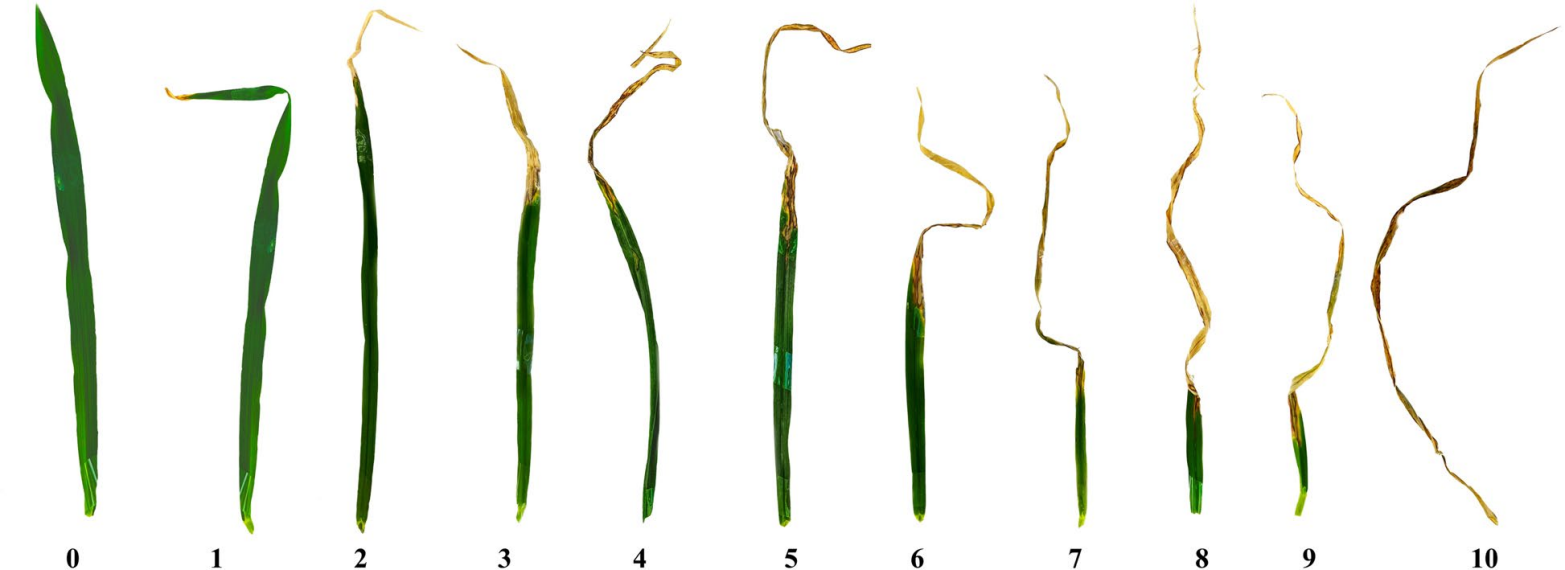
Scoring scales of *R. graminicola* infection in barley leaf with the increasing order of disease severity, 0 was asymptomatic and 10 was the highest severity of infection

The score ranged between 0 and 10, where 0 indicated no disease symptom occurrence, and 10 indicated the highest severity (Fig. 6), following the methods of [32]. The check cultivars were used to adjust the disease scores of the gene bank materials using the AugmentedRCBD package version 0.1.7 in R 4.2.2 [33]. With these adjusted means (disease scores), the area under the disease progression curve (AUDPC) was subsequently calculated using the same statistical package for individual experiments.

$$\text{AUDPC}= \sum_{\text{i}=1}^{\text{n}-1}(\frac{{\text{y}}_{\text{i}+}{\text{y}}_{\left(\text{i}+1\right)}}{2} )({\text{t}}_{\left(\text{i}+1\right)}-{\text{t}}_{\text{i}})$$

where y_i_ is the score at the i^th^ observation, t_i_ is the time (DAI) at the i^th^ observation, and n is the number of observations. The resulting AUDPC values from the two experiments were used to calculate the best linear unbiased estimator (BLUE) using META-R 6.04 software [34].

$${\mathrm y}_{\mathrm{il}}=\mathrm\mu+{\mathrm G}_{\mathrm{il}}+{\mathrm R}_{\mathrm l}+{\mathrm\varepsilon}_{\mathrm{il}}$$

where y_il_ is the AUDPC of the i^th^ genotype in the l^th^ replicate, µ is the general mean value, G_il_ is the i^th^ genotype effect in the l^th^ replicate, R_l_ is the effect of the l^th^ replicate, and ε_il_ is the residual effect.

The broad-sense heritability (H^2^) was calculated using META-R 6.04 software [34].

$${\text{H}}^{2}= \frac{{\upsigma }_{\text{g}}^{2}}{{\sigma }_{\text{g}}^{2}+{\sigma }_{\text{e}}^{2}}$$

where H^2^ is the broad-sense heritability and ${\upsigma }_{\text{g}}^{2}$ and ${\upsigma }_{\text{e}}^{2}$ indicate the genotype and error variance components, respectively.

### Genotyping, linkage disequilibrium, and genome-wide association analysis

The tested barley accessions were genotyped using the 15 K Illumina Infinium array from TraitGenetics GmbH (SGS, Germany). Single nucleotide polymorphism (SNP) markers with more than 10% missing values were removed from the analysis, refining the dataset to 13,268 markers, and after setting the minor allele frequency (MAF) threshold at 0.05, a total of 10,151 markers remained for the GWAS. The physical position of the genetic markers were determined following the Morex V3 reference genome [35]. The list of markers and the genotypes used for GWAS are provided in Supplementary Table S5. Two multi-locus models, Bayesian-information and linkage-disequilibrium iteratively nested keyway (BLINK) [36] and FarmCPU (fixed and random model circulating probability unification) [37] from GAPIT 3 [38], were used to detect markers associated with scald incidence. The threshold for identifying significant markers was set at a *P* value ≤ 0.05 with Bonferroni correction (0.05/number of markers), attaining -log10 (0.05/10,151) = 5.3 with a *P* value of 3.77e^−6^.

The squared correlation coefficient (*r*^*2*^) was calculated at the chromosome-wide and genome-wide linkage disequilibrium (LD) levels in TASSEL [39] using a sliding window of 50 SNPs [40]. A total of 10,151 markers were used to estimate LD. The calculated* r*^*2*^ was plotted against the physical distance (i.e., base pairs) between the locus pairs by adding a smoothing spline regression line, and individual chromosome-wide LD decay curves and genome-wide LD decay curves were generated in R 4.2.2 using the genetics package version 0.1.3 [41].

The QTL were identified based on the LD decay, meaning significant detected marker-trait association (MTAs) beyond the chromosome-wide LD decay were considered likely to segregate independently, suggesting different loci influencing the disease resistance [42]. We followed the same strategy for QTL validation. We initially performed a literature search to understand if any QTL reported in previous studies were located within the LD decay distance with the identified QTL in the current study. If we found no QTL, we considered the currently identified QTL a unique one.

### Favorable allele identification

The allelic effects of the detected MTAs from the GWAS were analyzed to observe their impact on scald resistance. First, a linear model was used with BLUE as the response variable and the detected MTA from the GWAS as the categorical predictor. Then, the least-squares means of the alleles of each MTA were calculated using the emmeans package (version 1.10.1). Subsequently the cld (compact letter display) function was employed in the multcomp package (version 1.4–25) to summarize and interpret the differences in BLUE according to the allele (the results are shown in Supplementary Table S2, S6 and S7). The results were then visualized using the ggplot2 package (version 3.5.1) in R 4.2.2. MTAs with significant allelic effects were selected, and their combination effects on the BLUE values were observed.

## Discussion

This study analyzed the resistance of 275 spring barley germplasm with four check cultivars that had different levels of scald resistance using phenotypic data collected under greenhouse conditions. The analysis revealed a significant phenotypic variation among the tested germplasms and a heritability estimate of 0.73.

In addition to heritability, linkage disequilibrium (LD) analysis helps the breeders identify the genomic regions linked to plant disease resistance more accurately [43]. The current study identified the LD decay at the chromosome and whole genome levels (1.4–4.4 Mbp across seven chromosomes and 2.1 Mbp for the whole genome) using 10,151 SNP markers. Many studies have reported a different range of LD decay associated with barley scald disease, each associated with different SNP densities. For instance, an association mapping study using 316 spring barley genotypes reported the LD decay of ~ 600 kb while using 36,793 SNP markers and identified 15 QTL associated with seedling stage scald resistance [44]. Similarly, another association study with a barley core collection (298 elites and 812 plant genetic resources) observed an LD decay of 2 Mbp while using 57 million SNP markers and identified 22 QTL [45]. Interestingly, some studies used much higher SNP densities and reported a higher LD decay. For example, a barley MAGIC population study using 27,407 SNP markers reported an LD decay ranging from 7 to 19 Mbp across seven chromosomes, detecting six QTL associated with scald resistance [46]. A shorter LD decay is beneficial because it enhances the precision of genomic regions associated with the trait and assists in developing a more efficient breeding strategy [47]. Even with a lower SNP density, the LD decay in our study captured important genetic variations, highlighting the efficiency of our approach in identifying important genomic regions associated with scald resistance.

Among the three QTL identified on chromosome 3H, QTL_3H_1 is likely a potential new QTL identified for scald resistance. This QTL is located at 424.23 Mbp on Chr 3H, and the nearest QTL previously identified is QTLR3H.4 (428.8 Mbp). This QTL was identified as a scald-resistant QTL in double haploid (DH) mapping populations of winter barley (a population developed between Saffron, the susceptible cultivar, and Retriever, the cultivar resistant to scald) The author considered that QTLR3H.4 overlapped with the *Rrs1* locus, affecting 30% of the phenotypic variance of their tested barley population. The distance (4.6 Mbp) between QTLR3H.4 and QTL_3H_1 from the current study exceeds the observed LD decay (2.3 Mbp) at Chr 3H, suggesting that the linkage between these QTL is not strong enough and has a chance of being separated during segregation. Another QTL, QTL_3H_2, was found only 490 kb away from *Rrs. B87 was* identified in the B87/14 spring barley line [48]. T The author considered that QTLR3H.4 overlapped with the *Rrs1* locus, affecting 30% of the phenotypic variance of their tested barley population. The distance (4.6 Mbp) between QTLR3H.4 and QTL_3H_1 from the current study exceeds the observed LD decay (2.3 Mbp) at Chr 3H, suggesting that the linkage between these QTL is not strong enough and has a chance of being separated during segregation. Another QTL, QTL_3H_2, was found only 490 kb away from *Rrs.B87 was* identified in the B87/14 spring barley line [49]. This gene is considered a single dominant gene against *R. graminicola* located close to the well-known *Rrs1* complex locus [49]. Avr*Rrs1* triggers the resistance mechanism by recognizing the necrosis-inducing protein 1 (NIP1) fungal protein via a resistant host plant carrying the *Rrs1* gene [50]. Additionally, it was reported that *Rrs1* suppresses the growth of fungal hyphae and leads to the formation of a fungal network that grows randomly in different directions instead of forming a functional fungal network in Atlas 46, a resistant cultivar that possesses the *Rrs1* gene [51]. Another QTL, QTL_3H_3 (450.44 Mbp), located at 449.9 Mbp, was found to be proximate to QTLCB3H.4, which was previously identified in the Spanish landrace-derived line SBCC145 [52]. In addition, several QTL contributing to scald resistance have been detected on Chr 3H in various spring barley collections. These genes are located at 447.3 Mbp, 454.9 Mbp, and 455.3 Mbp on Chr 3H [10, 11, 52–54]. Possible explanations for the presence of multiple QTL related to scald resistance along Chr 3H have been proposed as follows: (1) *Rrs1* is a complex locus containing many alleles of the same R gene; (2) *Rrs1* is a set of closely connected genes; or (3) *Rrs1* is a combination of both [11].

Another significant QTL detected in the current study is QTL_6H, which is also considered a new QTL for scald resistance. Studies have identified 30 QTL related to barley scald resistance on Chr 6H with a total physical distance equivalent to 580.00 Mbp [11, 16, 20, 21, 48, 54–59]. Among these QTL, Qsc3.6H.7-Seebe (545.5 Mbp) [54], is the closest QTL to QTL_6H detected in the present study, despite the considerable distance of 34.8 Mbp between them (510.7 Mbp vs. 545.5 Mbp). Notably, most of these QTL (25 out of 30 QTL) were detected at the proximal region of Chr 6H, while only 5 QTL were identified at the distal end of the chromosome. Although the location of the QTL, whether proximal or distal, may not directly impact the phenotype, identifying new QTL at the distal end where the QTL has been sparse contributes to the diversification of genetic factors known to influence scald resistance in barley.

Four MTAs were detected on Chr 7H via GWAS, and three QTL were identified. Among the four MTAs, two (BOPA2_2_20201 and JHI-Hv50k-2016–441289) were located close to each other (only 820 kilobase pairs apart) and therefore considered the same QTL (QTL_7H_1). At the same time, this QTL was found to be near the previously known QTL *Rrs2*. One of the earliest reports related to *Rrs2* [60] reported that in Digger (the resistant cultivar), halos were formed in the cell walls, and larger papillae were produced than in Osiris (the susceptible cultivar), despite the identification of the causal gene mediating such subcuticular modification. Later, *Rrs2* was fine-mapped in the F_2_ population derived from a cross between Atlas (the resistant cultivar) and Steffi (the susceptible cultivar), and the physical position of *Rrs2* was detected at 5.2 Mbp [13]. This *Rrs2* gene overlaps with QTL_7H_1 from our study.

QTL_7H_2 was closely located to the previously reported QTL, *Rh2,* which is located at position 10.8, and this QTL was detected in the Atlas, a scald-resistant spring barley cultivar [61]. To our understanding, QTL related to other major barley diseases are also located at the end of the 7H short arm region, such as Rdg2a for barley leaf stripe [62] and Rpg1 for barley stem rust [63] near QTLTritonRrs7H271, making this region of Chr 7H worthy of further evaluation to identify potential scald-resistant loci. Furthermore, QTL_7H_3 is a new locus identified in the current study, and it is located at 621.6 Mbp on Chr 7H. Its nearest known QTL is *Rrs1*5 (626.3 Mbp) [18], which is located at a distance of 4.7 Mbp from QTL_7H_3. *Rrs1*5, derived from the Israeli accession of wild barley, was successfully mapped in the third backcross population between that accession and a scald-susceptible cultivar called Clipper [18].

Two genotypes, AKKA and FRIDA possessed three favorable alleles each. However, the presence of an unfavorable allele of one of the MTAs detected at QTL_7H_1 (relevant MTA: JHI-Hv50k-2016–441289) in FRIDA reduced resistance, resulting in a higher BLUE value. In contrast, for AKKA, the favorable alleles from both MTAs at QTL_7H_1 likely worked synergistically to enhance resistance, even without the favorable allele from QTL_3H_1. Remarkably, the favorable alleles of QTL_3H_1 and QTL_7H_1 (relevant MTA: JHI-Hv50k-2016–441289) were found together only in the genotype Solar, which possesses all the favorable alleles. A study reported that the presence of favorable alleles across different QTL can enhance resistance. However, the combinations of all these QTL may rarely occur in different genetic backgrounds [64]. Twenty genotypes with two favorable alleles from either two of the significant MTAs showed further decrease in resistance, with an average BLUE of 34.61. Based on the results from haplotype comparisons (Supplementary Table S3), the BLUE values of these haplotypes were not statistically different. They are still more resistant than those with a single allele (with an average BLUE of 42.05) and those without any favorable allele (with an average BLUE of 44.1). Thirty-two genotypes had a single favorable allele from one of the significant MTAs. Following the results from the haplotype comparisons, there was no significant difference between the average BLUE values of the genotypes with one favorable allele and those with no favorable allele. However, when examining the individual genotype, two genotypes (MENTOR and KORU) with only one favorable allele from the MTA, BOPA1_1977-1385 had higher BLUE values than the susceptible checks with no favorable allele. The only favorable allele presented in MENTOR and KORU was located at the QTL_3H_1 region near the *Rrs1* complex locus. However, this QTL alone could not overcome the unfavorable alleles'strong collective effect. Another possibility is that these unfavorable alleles interact with other unfavorable alleles in other genomic regions, dominating the favorable allele effect.

In summary, identifying new QTL enhances our understanding of scald resistance in barley and highlights potential targets for future breeding programs. The discovery of novel loci and their relationships to known resistance genes provides valuable insights into the genetic architecture of disease resistance, which could be leveraged to develop more resilient barley cultivars. Research focusing on fine-mapping candidate genes in these detected QTL regions should be conducted to identify the causal variants for scald resistance. While these types of genetic variants can be discovered and evaluated under controlled environmental conditions, field experiments should be carried out not only considering the effects of environmental conditions and their interactions but also considering the stability of the effects of QTL across both space and time, thereby strengthening the resulting breeding strategies that were identified in this research.

## Conclusions

The current study utilized association mapping analysis to examine the genetic variation associated with scald disease in 279 spring barley genotypes. The significant genetic variation observed among the barley germplasms highlights the abundant genetic diversity within the tested gene bank germplasm. This diversity is valuable for improving crop performance and resilience in breeding programs. Furthermore, our study revealed three novel QTL that offer fresh insights into the genetic basis of resistance traits and three previously identified QTL, reinforcing the robustness and reliability of this investigation. Additionally, MTAs with favorable effects on disease progression could be valuable for enhancing barley scald resistance through marker-assisted breeding targeting the development of barley varieties with strong resistance, underscoring the importance of ongoing research programs. Additionally, future studies, such as identifying candidate genes within these QTL and developing KASP markers for breeding programs targeting improving scald resistance and enhancing crop productivity through precision breeding strategies, are suggested.

## Supplementary Information

Supplementary Material 1.

Supplementary Material 2.

## Data Availability

The data that support the findings of this study are provided in the additional files.
