# Supplementary material for: Harnessing novel genetic markers for scald resistance from gene bank spring barley genotypes
Source: BMC Plant Biol. 2025 Jun 11;25:781. doi: 10.1186/s12870-025-06813-2 (PMC12160430; doi:10.1186/s12870-025-06813-2)
Supplement: Supplementary file 1 — Supplementary Material 1. [file 12870_2025_6813_MOESM1_ESM.docx]

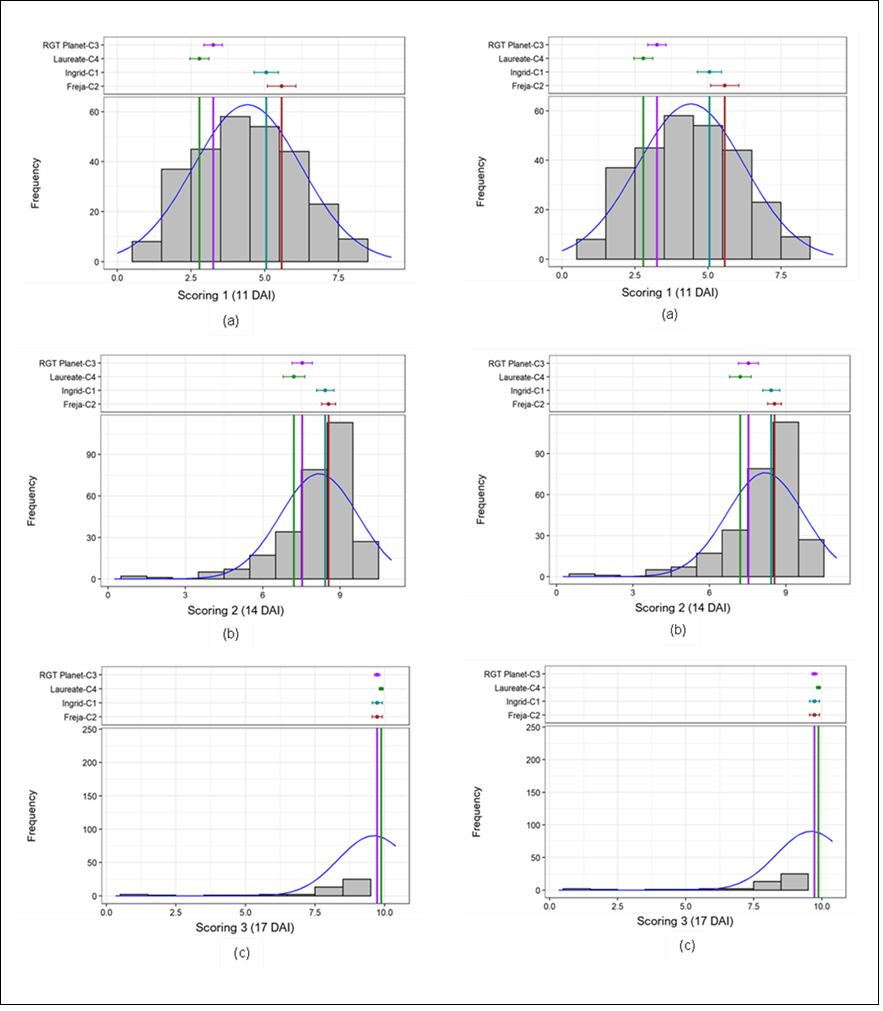


Supplementary Figure 1. Frequency distribution of the tested barley genotypes in trial set 1 (left panel) and trial set 2 (right panel). (A) first scoring at 11 DAI; (B) second scoring at 14 DAI; (C) third scoring at 17 DAI


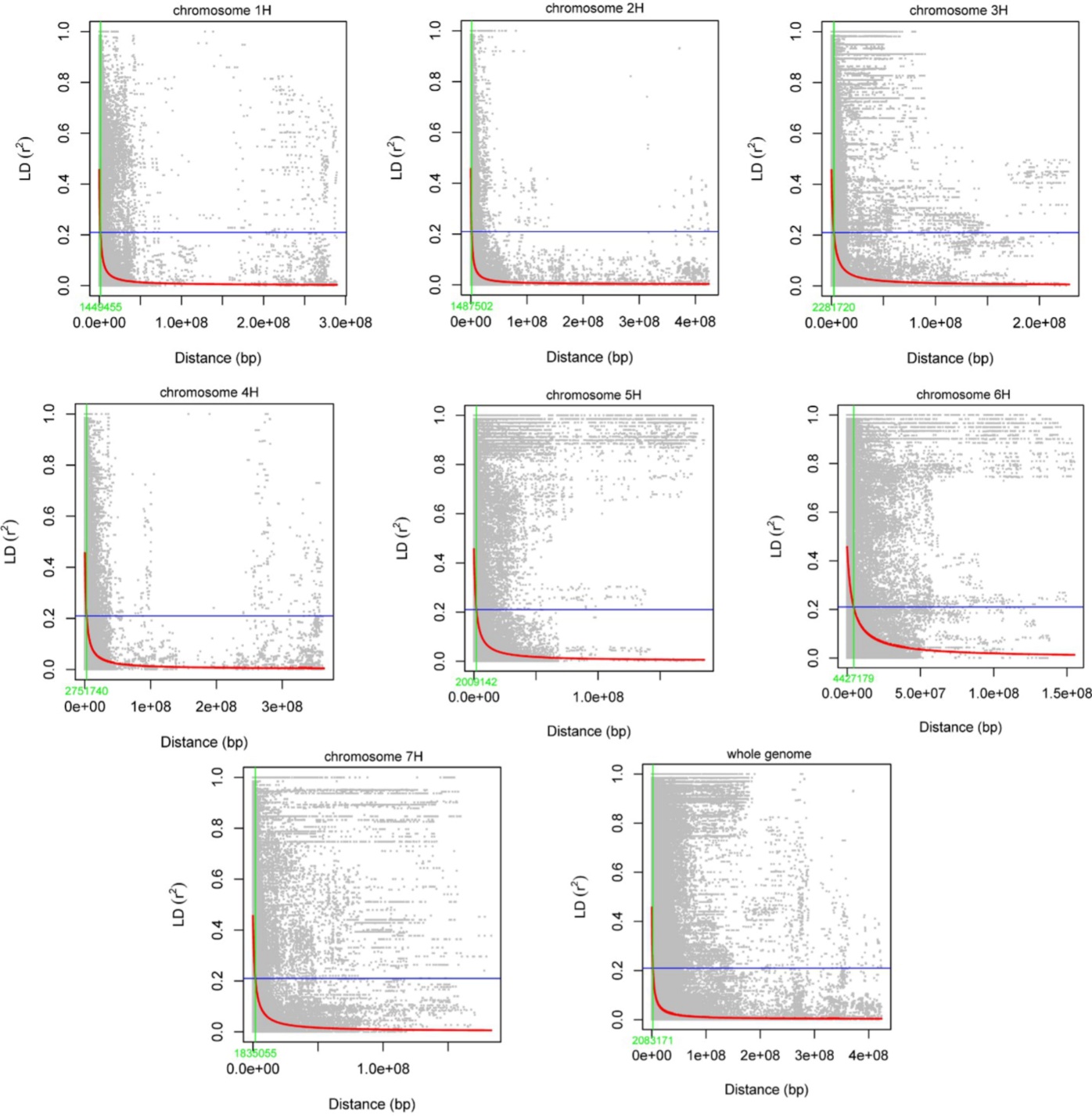


Supplementary Figure 2. Chromosome level LD decay curve fitted into a scatter plot with r^2^ values against the physical distance (bp). The red curve line in each figure was the smoothing spline regression model fitted to LD decay for each chromosome. The AUDPC (BLUE) horizontal line showed the half decay r^2^ value and the green vertical line represented the distance between the marker pairs where at the intersection between the half decay and the LD decay curve.


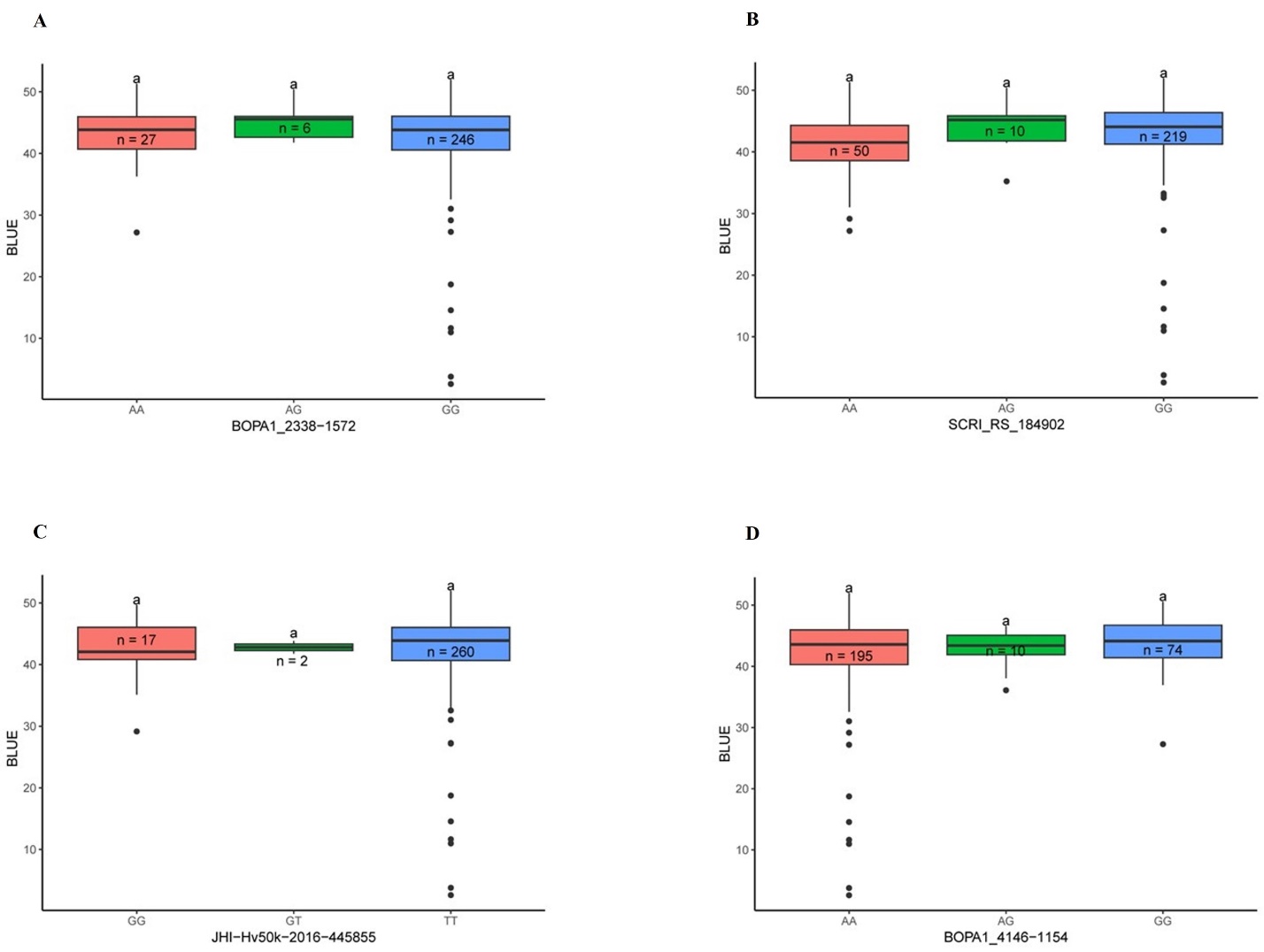


Supplementary Figure 3. Boxplots illustrating the allelic effect of SNPs detected from GWAS. BOPA1_2338-1572 (A), SCRI_RS_184902 (B), JHI-Hv50k-2016-445855 (C) and BOPA1_4146-1154 (D). Tukey's HSD (honestly significant difference) test was performed to check the significance level by comparing the allelic composition of the barley germplasms.
